# Bridging global health actors and agendas: the role of national public health institutes

**DOI:** 10.1057/s41271-022-00342-0

**Published:** 2022-04-12

**Authors:** Sonja Myhre, Mahlet Kifle Habtemariam, David L. Heymann, Trygve Ottersen, Camilla Stoltenberg, Deisy de Freitas Lima Ventura, Eirik F. Vikum, Anne Bergh

**Affiliations:** 1 Norwegian Institute of Public Health, Oslo, Norway; 2 Africa Center for Disease Control (CDC), Nairobi, Kenya; 3 London School of Tropical Medicine, London, UK; 4 Chatham House Centre for Universal Health, London, UK; 5 University of São Paulo, Av. Dr. Arnaldo, 715, São Paulo, SP Brazil

**Keywords:** National public health institute, Health systems, Health security, Governance

## Abstract

Global health crises require coordination and collaboration among actors and global health agendas including health security, health promotion, and universal health coverage. This study investigated whether national public health institutes (NPHIs) unify agendas and actors, how this can be achieved, and what factors contribute to success. We conducted semi-structured interviews with 24 public health leaders from 18 countries in six WHO regions between 2019 and 2020. Respondents described how NPHIs bridge agendas reporting five strategies that institutes employ: serving as a trusted scientific advisor; convening actors across and within sectors; prioritizing transdisciplinary approaches; integrating public health infrastructures, and training that builds public health capacity. Findings also revealed five enabling factors critical to success: a strong legal foundation; scientific independence; public trust and legitimacy; networks and partnerships at global, national, and local levels; and stable funding. The Covid-19 pandemic underscores the urgency of securing scientific independence and promoting national institutes’ responsiveness to public health challenges.

**Supplementary Information:**

The online version contains supplementary material available at 10.1057/s41271-022-00342-0.

## Key messages

- Scientifically independent national public health institutes can serve an important role by bridging health agendas and unifying diverse actors.
- National public health institutes should serve as trusted scientific advisors.
- A legal foundation, scientific independence, political will, public trust, partnerships, and funding can facilitate and ensure success.

## Introduction

The COVID-19 pandemic ushered in a new era of public health around the world. As of February 2022, 220 countries and territories have reported more than 460 million confirmed cases and over 6 million deaths [1]. The devastating impact of this pandemic has been compounded by severe economic repercussions further underscoring the world’s shared vulnerability. Public health emergencies of international concern demand coordination and collaboration among multiple agendas and actors including those central to the World Health Organization’s (WHO) triple billion goals: health security, universal health coverage, and health promotion. WHO’s aim is to ensure one billion more people benefit from universal health coverage, one billion more are better protected from health emergencies, and one billion more improve their health and well-being [2]. Moving towards these targets by 2023 and advancing work on the Sustainable Development goals will depend on individual countries’ ability to address all three agendas. In resource-constrained settings, it is even more imperative that solutions are synergistic across agendas.

This study is based on the premise that countries must invest in health development that enables simultaneous progress on several agendas to achieve these ambitious goals [3]. No studies to date have investigated whether national public health institutes (NPHIs) bridge agendas and unify actors, how this can be achieved, and what factors contribute to success. By ‘bridge’, we mean bringing together and synergizing these global health agendas. This study examines these questions, made even more salient by the Covid-19 pandemic, which underscores the need for strong national public health coordination and collaboration. The aim of this study is to explore whether and how NPHIs may further bridge global health agendas and actors and what factors make this possible.

## The role of NPHIs

This study examines NPHIs for several reasons. NPHIs are increasingly recognized for their contributions during health emergencies [4, 5] and tackling enduring national and global health challenges [6]. Previous experience from SARS and Ebola led to investment in creating NPHIs or merging existing agencies into single consolidated entities. In the aftermath of SARS, for example, Hong Kong and Canada established NPHIs in response to national recommendations citing the need for improved coordination, national guidance, and leadership [7, 8]. Similarly, Liberia and Sierra Leone created agencies to lead their country’s public health efforts after the 2014 Ebola outbreak [9, 10]. Several publications have made the case for building capacity through investments in national public health institutes to strengthen nationally owned and led responses to epidemic preparedness [11–13]. COVID-19 may rekindle this interest.

## Definitions of concepts

### National public health institutes

The International Association of Public Health Institutes (IANPHI) defines a NPHI as “a government agency, or closely networked group of agencies, that provides science-based leadership, expertise, and coordination for a country’s public health activities.” NPHIs have a longstanding legacy as politically neutral, semi-autonomous, and in many cases also explicitly scientifically independent governmental agencies supporting Ministries of Health (MoH) undergirded by legal frameworks. NPHIs around the world use many different nomenclatures (for example, institute, center, agency, center for disease control) reflecting historical origins, language, and governance traditions. Legal status and organizational structure also reflect country context, yet the common thread is a dedicated focus on core public health functions [14].

### Universal health coverage, health security, and health promotion

Given the broad range of definitions associated with the three global health agendas, and while recognizing there is no consensus, we define these terms for the purpose of this study as follows:

**Universal health coverage:** access to quality essential health care services including medicines, vaccines, and financial risk.
**Health security:** activities needed to reduce vulnerability of risks to health, particularly those that can potentially spread across international borders.
**Health promotion:** activities that entail population-based interventions to promote healthier lives and well-being.

## Methods

### Study design

The study team conducted semi-structured interviews with 24 public health leaders with extensive expertise, experience, or knowledge of national public health institutes from 18 countries in six WHO regions. We analyzed the interview data using an inductive content analysis approach to identify themes from the interview collection. We integrated principles derived from the COnsolidated criteria for REporting Qualitative research (COREQ) from the outset in the study design, data collection, and reporting process [15]. Supplemental material contains a completed COREQ checklist (Table S1), interview information sheet (Table S2), informed consent form (Table S3), and interview guide (Table S4).

### Key informant interviews

We derived a formative list of key informants from a scoping review of NPHI literature [16]. We conducted initial interviews with twelve authors of peer-reviewed journal articles focusing on NPHIs. Using a snowball recruitment strategy, we asked interviewees to recommend highly qualified candidates. Consultation with co-authors and a purposive strategy guided informant selection to attain representation by gender, geographic location, organizational maturity, size, and country-level economic status. We contacted potential candidates by email and invited them to participate. We did not reject or exclude any potential informants from participation although some were unresponsive despite several email follow-up attempts. We chose selection criteria to include perspectives from single NPHIs, networked agencies, and country settings without designated NPHIs. We conducted interviews by teleconference (n = 19) and face to face (n = 5). Before the interview, we sent participants a project summary, informed consent form, and list of questions (see Appendix 2). Two authors (SLM and AB) with previous interview experience conducted the interviews, all in English except two using interpreters. We considered issues related to researcher reflexivity in advance. We conducted interviews from November 2019 to February 2020. According to the project timeline, we conducted no interviews after February. The first interview pilot test of the interview guide resulted in minor modifications. We audiotaped and transcribed all interviews. Participants received transcript copies and were provided an opportunity to correct them. Interview length ranged from 30 to 75 min.

### Data analysis

For qualitative analysis of the interview data, we used a thematic analysis approach described by Braun and Clarke [17] that entails identification of cross-cutting themes and patterns without relying on preconceived ideas or categories to ensure openness to new concepts [17]. Based on an inductive qualitative data analysis approach, the process involved compiling the data via transcription; coding data into discreet units; exploring codes for patterns and creating broader categories; and crafting broad themes to capture overarching concepts. Several authors discussed thematic categories and supporting codes to reach consensus.

### Ethics

We designed the study in accordance with the General Data Protection regulations and submitted study documentation to the Norwegian Institute of Public Health that approved it. Given that participants’ identities were confidential and anonymous, the study did not require further ethical review.

## Results

This section describes the informant sample; participants’ views about NPHI contributions to health security, universal health coverage, and health promotion; strategies used by NPHIs to bridge global health agendas; and key enabling factors critical to NPHI success.

### Key informant sample

We included key informants based in all six WHO-defined geographic regions (Africa, the Americas, Europe, Eastern Mediterranean, South East Asia, and the Western Pacific) who worked in the following countries: Burkina Faso [2], Brazil [3], China [1], Ethiopia [1], Finland [1], India [1], Mali [1], Mexico [1], Morocco [2], Mozambique [1], Netherlands [1], Nigeria [2], Palestine [1], Republic of Moldova [1], Sierra Leone [1], South Africa [2], Ukraine [1], and the US [1]. Twenty-one informants were male and three were female.

Informants in the sample had extensive knowledge of NPHIs. Seventeen informants had been acting or former Director or deputy Director of a NPHI or similar agency. Several had worked to establish their NPHI. Others had professional experience in academia or held a leadership role in a regional or global public health organization. The sample also included individuals with experience in different size NPHIs including large (1000 + staff), medium (101–999), and small (less than 100) agencies. Similarly, experience with NPHIs included mature (20 + years), established (6 to 19 years old), and newly created NPHIs (5 years or fewer). To preserve anonymity, we refer to comments expressed by informants by number in parentheses (i.e., #1–#24). We present illustrative quotes from key informants in Table S5 in the Supplemental Material and referred to in parentheses (for example, Table S5, line #, informant #).

### NPHIs’ contributions to global health agendas

While many informants acknowledged the interdependence of the agendas, several informants commented that WHO’s definitions of health security, universal health coverage, and health promotion are often not understood at local and national levels (#1, #5, #14, #16). Others remarked that this terminology may not resonate with national views (#13, #17, #19). Conceptual overlap made it challenging to pigeonhole NPHI functions into categories as noted by one informant (Table S5, line 1, #5).

#### NPHIs and health security

The legacy of NPHIs’ historic roots in sanitation, laboratories, hygiene, and outbreak detection is still evident in their prevailing health security focus. According to informants, most NPHIs prioritize health security efforts through several core functions such as disease surveillance, laboratory diagnostics, investigation of health threats, emergency preparedness, and response. Many NPHIs engage in activities related to the International Health Regulations (IHR) and Joint External Evaluations as part of national health security efforts (#1, #7, #10, #12, #24). Specific examples include efforts dedicated to development of National Action Plans for Health Security or aimed at strengthening biosafety and biosecurity in support of the Global Health Security Agenda (#8, #11, #12, #24).

#### NPHIs and universal health coverage

Many informants indicated that MoHs or other state agencies often spearhead universal health coverage efforts (#1, #2, #9, #10). Although one informant reported that universal health coverage was the primary focus, all others mentioned indirect involvement through promotion, surveillance, or research activities. Several noted that focus on fiscal mechanisms can overshadow attention given to quality or service-oriented components (#2, #5, #16). One observed overlap among agendas for planning purposes (Table S5, Line 2, *#1*).

#### NPHIs and health promotion

Informants’ responses about health promotion reflect a broad range of approaches. A few indicated the top priority to be health promotion (#7, #15) while several considered this the weakest pillar (#2, #12, #21). Other informants commented on the importance of this agenda to reduce demand for services (#4, #22). Several cited generating relevant evidence as an example of NPHIs’ indirect contribution to health promotion. National HIV survey data, for example, revealed decreasing levels of knowledge that led governments and funders to renew their investments in promoting healthy behaviors (#12). Another theme, tackling social determinants, underscored the cross-sectoral nature of public health (Table S5, line 3, #10).

### Strategies for bridging global health agendas

This section describes respondents’ insights into how NPHIs have successfully bridged global health agendas. Respondents articulated five actionable strategies NPHIs have used to bridge agendas, namely serving as a trusted scientific advisor to inform policy, priorities, and decision making; convening actors across and within sectors; prioritizing multi-, inter-, and transdisciplinary areas; developing public health capacity through teaching and training; and integrating public health infrastructure for multipurpose use. Table 1 provides a summary of these strategies, country examples, and resulting links across agendas.

**Table 1:** Actionable strategies, country examples, and resulting linkages across agendas

Actionable strategies	Examples at the country level	Resulting linkages across agendas
Serve as trusted scientific advisor to inform policy, priorities, and decision making	The scientific independence of NPHIs ensures that sharing knowledge in the form of, for example, research or data from registries, surveys, disease surveillance systems, systematic reviews, and health technology assessments will build trust and credibility and inform policy, priorities, and decision making	The common ground of science-based advice that can inform policy, priorities, and decision making facilitates knowledge linkages across all three agendas
Convene actors across and within sectors	Health security efforts via IHR coordination convenes multiple intersectoral actors across ministries and external stakeholders; Tackling infectious disease, outbreaks, and non-communicable disease also requires intersectoral cooperation among experts from medicine, education, sanitation, housing, ecology, economics and local municipalities to mobilize resources, activities and expertise	Intersectoral convenings and consensus-building processes create interlinkages across health security, universal health coverage and health promotion agendas
Prioritize multi, inter-, and transdisciplinary approaches	Tackling obesity requires multidisciplinary, multisectoral, multilevel cooperation; scientific input from nutrition, medicine, education, marketing, law, and policy contributes to policies, school guidelines, labeling, and tax policy. One Health, tobacco control, and antimicrobial resistance are other examples	Linkages across diverse disciplines, sectors, and levels (local, national, global) fortifies links between health promotion with universal health coverage
Integrate public health infrastructure for multipurpose use	Integrated public health infrastructure including laboratories, data information systems and disease surveillance platforms for multipurpose use has the potential to reduce duplication, waste, and inefficiency and promote better understanding of comorbidity trends across diseases	Linkages across public health infrastructure supporting universal health coverage (e.g., HIV/AIDS, tuberculosis surveillance systems) and health security (e.g., infectious disease identification)
Develop public health capacity through teaching and training	NPHIs in countries including Brazil, China, Mexico, Mozambique, Nigeria, and South Africa, for example, offer field epidemiology training courses, or professional education including master’s and doctoral postgraduate coursework or informal training that, in turn, strengthens the public health workforce and institutional capacity	Public health capacity building through education and training links all three agendas and contributes to broader public health competencies and skill sets

#### Serve as trusted scientific advisor to inform policy, priorities, and decision making

Informants acknowledged that NPHIs’ unique position of scientific independence facilitates serving as a credible, trusted advisor to the MoH, the government, and the general public (#6, #10–14, #16, #18, #20, #23). As science-based agencies, NPHIs gather evidence and generate data that, in turn, lends credibility to policy decisions that contribute to building public trust (Table S5, line 4, #16). Informants also mentioned the value of data and health information systems as building blocks of research and catalysts for decision making (#4, #6, #11, #12, #18, #21, #23). NPHIs can collectively add value by collecting and working with data (Table S5, line 5, #14). Similarly, an informant commented more generally on the challenge of prioritizing investment in research (Table S5, line 6, #22).

#### Convene actors within and across sectors

Informants often mentioned the intersectoral convening role orchestrated by NPHIs (#3, #6, #7, #10, #12, #21, #23, #24). One informant described a NPHI-driven initiative involving a multisectoral consensus-building process to develop a National Action Plan for Health Security (#12). Several ministries and external stakeholders engaged throughout the process resulting in consensus building, buy-in, and ownership (Table S5, line 7, #12). Adapting the process to their cultural context resulted in an instrument with relevance and national value. They incorporated elements from health promotion and universal health coverage into the document underscoring the added value of multisectoral cooperation. Other informants described situations having involved convening diverse stakeholders from health, food safety, animal, environment, and economic agencies to address issues such as antimicrobial resistance (#7), listeria (#16), and dengue (#3).

#### Prioritize multi-, inter-, and transdisciplinary approaches

Given the complexity of many public health challenges, multi-, inter-, and transdisciplinary approaches are critical (#3, 10, #17, #18, #22). One informant provided addressing obesity as an example of integrating knowledge from several disciplines into a holistic approach (#3). Epidemiologic studies identified national obesity trends leading to research investigating social and environmental drivers of obesity. Economic surveys and analysis of consumer databases on food expenditure and entertainment revealed a dramatic rise in soda consumption and decline in healthy foods. Presentation of this research to the MoH led to multilevel, multisectoral, and multidisciplinary interventions such as school guidelines, food labeling, adoption of national policy documents, and a sweetened beverage tax illustrating the value of integrating approaches.

#### Integrate public health research infrastructure for multipurpose use

Several informants described the potential of NPHIs to facilitate integration of public health infrastructure for multipurpose use (#3, #4, #11, #13, #17, #20). As one informant observed, in resource-constrained settings, it is essential to maximize efficiency and minimize disease-specific approaches (Table S5, line 8, #11). Such a strategy may remedy vertical programming’s parallel systems, wasted resources, and missed opportunities. (Table S5, line 9, #11). In sum, informants describe NPHIs as well positioned to advocate for building platforms, pooling activities, and sharing resources*.*

#### Develop capacity through teaching and training

Many informants mentioned developing public health capacity through teaching and training as an integral part of their NPHI’s mission (#3, #6, #7, #12, #13, #14, #15, #18, #23). Respondents reported various types of educational opportunities including field epidemiology training courses, professional training programs, or postgraduate coursework. Brazil and Mexico have adopted a unique approach to building public health education systems by establishing accredited schools of public health programs, each under the umbrella of their NPHI with the explicit aim of developing the national public health workforce (#3, #13). Building educational opportunities into the ethos of NPHIs may reduce brain drain, repatriate talent of individuals studying abroad, consolidate expertise, provide inspiring career tracks, and stimulate local research activities (#18).

Findings gleaned from interview data suggest that NPHI-initiated strategies depend on key enabling factors to thrive as described in Table 2. Factors facilitating success clustered around five themes, namely a strong legal foundation with a multidisciplinary mandate; political will and acceptance of scientific independence; public trust and legitimacy; networks and partnerships at global, national, and local levels; and stable funding.

**Table 2:** Key enabling factors

Key enabling factors	Value and importance for sustainability
A strong legal foundation with a multidisciplinary mandate	Legal footing in the form of law, decree, act, or other legal means codifies authority and enforcement capability. Legal demarcation of NPHI’s multidisciplinary mandate ensures long-term engagement in complex public health challenges
Political will and acceptance of scientific independence	Institutional scientific neutrality safeguards credibility and ensures that NPHI advice and efforts are not silenced, sidelined, or scapegoated as a result of political partisanship or interference
Public trust and legitimacy	Built on organizational independence, public trust and legitimacy are achieved through perceived integrity, relevance, and value of NPHI work as well as credibility of scientific methods
Partnerships and networks at the global, national, and local levels	Sectoral cooperation in country and global and regional networks (Africa CDC, IANPHI) enhances efficiency through knowledge exchange, resource sharing, and collaborative work at global, national, regional, and local levels
Access to stable funding	Core, domestic from government budgets and external funding from bilateral donors, private foundations, or global institutions assures NPHI’s organizational longevity and sustainability

Nearly all respondents mentioned the necessity of a firm legal footing; failure to pass a law, act, or decree proved to be the principal deterrent in establishing a NPHI (#4, #10). The value of articulating a multidisciplinary mandate safeguards engagement in emerging public health challenges. Informants coupled a strong legal foundation with need for high-level political will to spearhead the process (#4, #5, #14, #18). They also deemed as critical for NPHI functionality, acceptance of scientific independence by politicians to avoid partisan interference (#13, #18, #20, #21, #23). Public trust and legitimacy rely on scientific independence as well as the credibility, integrity, and relevance of the work produced by NPHIs. Leveraging NPHI partnerships and networks on local, regional, and global levels indicates the importance of collaboration, particularly during crises. Finally, stable core funding from domestic budgets or external funds is essential for organizational sustainability and longevity (#1, #3, #14, #16, #18, #21).

## Discussion

Although the design and data collection of this study occurred before COVID-19, the findings are particularly relevant in the context of this pandemic. Country level struggles with COVID-19 continue to underscore the need for bridging agendas and actors. Countries with seemingly well-prepared health security infrastructure have, for example, performed poorly in terms of detection and death [18]. Lack of vaccines or curative treatments continues to reinforce the importance of health promotive efforts and universal health coverage to assure health care [19].

This pandemic has led some countries to re-examine their institutional capacity. Two countries have taken steps to establish NPHIs: Zimbabwe published a statutory instrument for a NPHI in July 2020 [20] and South Africa passed a law for a NPHI in August 2020 [21]. Observers have long recognized the value of NPHIs as health security focal points [22–24]. Engagement and support for universal health coverage and health promotion are consistent with research reporting that mature NPHIs, having accrued capacity and resources, adopt broader mandates with multiple agendas while fledging institutions have more limited scope that develops over time [25].

Our findings include several ways that NPHIs bridge global health agendas. Five actionable strategies demonstrate how NPHIs have drawn on their inherent core assets including their capacity for serving as a trusted scientific advisor to inform decision making; multisectoral convening; multidisciplinary approaches; multi-use infrastructure; and training. Regarding provision of scientific advice, the COVID-19 pandemic has shown how critical it is that NPHIs maintain scientific independence to provide data and evidence to inform decision making. Yet during this pandemic, examples may be found to the contrary where politically motivated actors have sidelined, suppressed, silenced, and scapegoated NPHIs, thus, exposing the tenuousness of scientific autonomy. Given that scientific independence forms the foundation of public trust [26], greater efforts will be needed to safeguard NPHIs’ ability to provide science-based advice to policymakers and the public. Scientific quality, integrity, and excellence are also vitally important in building public trust.

As to multisectoral convening, COVID-19 provides real-world lessons on the necessity of coordinating siloed sectors to address certain public health challenges. As impartial conveners, NPHIs are well suited to facilitate intersectoral cooperation. Similarly, the value of integrating disciplines and approaches continues to grow given the complexity of global health challenges such as climate change and antimicrobial resistance. The COVID-19 pandemic illustrates the need for multidisciplinarity as it has required scientific engagement from many different fields [27].

Integrating public health infrastructures for multipurpose use can be leveraged to increase efficiency, reduce waste, and optimize resources. Studies in countries that have integrated surveillance of HIV, tuberculosis, and sexually transmitted diseases have demonstrated that harmonized approaches that promote understanding of comorbidity trends across diseases reduce duplication and inefficiencies [28]. The urgency of NPHIs to access data sources for preparedness purposes raises similar issues on the value of ‘integrated science’ [29]. In sum, these examples demonstrate several unique strategies that NPHIs have used to bridge global health agendas. In resource-constrained settings, these strategies may be particularly useful in reducing fragmentation while also increasing collaboration, enhancing efficiencies through coordination, and instilling country ownership and stewardship.

The presence of several critical enabling factors is key for NPHIs to thrive. Governments should recognize their role in supporting NPHIs by providing a legal foundation with a comprehensive mandate, protections to ensure scientific independence that will instill public trust and legitimacy, and stable funding. Finally, regional and international NPHI organizations such as Africa Centres for Disease Control and Prevention and the International Association of National Public Health Institutes (IANPHI) continue to expand their membership benefits; these will be all the more valuable as information and resource-sharing opportunities advance [30].

### Limitations, strengths, and contributions

To our knowledge, no studies to date have investigated the unique contributions of NPHIs to WHO’s three global health agendas. Another strength of this study is the collection of diverse perspectives from high-level public health leaders from different regions around the world. A limitation of the study is the low representation from regions with few NPHIs (that is, Southeast Asia, Western Pacific). Other limitations concern the key informant recruitment process. While the selection process aimed to gather views from different geographic regions and institutional perspectives (staff size and NPHI maturity), using a snowball strategy may introduce selection bias by inadvertently acquiring individuals with similar beliefs and views. In addition, under-representation of women in our sample suggests that men often hold NPHI leadership positions in LMIC settings. Future research should investigate further perspectives from public health leaders in LMICs that have not established NPHIs.

## Conclusion

The study findings indicate that NPHIs bridge global health agendas and unify actors using strategies often associated with NPHIs. Further, these findings support investing in establishing new or strengthening existing NPHIs, given the potential value to countries. In LMICs, NPHIs offer a compelling case for investment using a country-owned approach that can maximize synergies across global health agendas, respond to pandemic situations, and contribute to a wider systems approach. Governments and other stakeholders, however, should carefully consider essential enabling factors that will facilitate success. The COVID-19 pandemic amounts to an urgent call to take bold steps to secure scientific independence and promote national institutions responsive to present and future public health challenges.

## Supplementary Information

Below is the link to the electronic supplementary material.

Supplementary file1 (DOCX 34 kb)
