# Supplementary material for: Bridging global health actors and agendas: the role of national public health institutes
Source: J Public Health Policy. 2022 Apr 12;43(2):251–65. doi: 10.1057/s41271-022-00342-0 (PMC9002032; doi:10.1057/s41271-022-00342-0)
Supplement: Supplementary file 1 — Supplementary file1 (DOCX 34 kb) [file 41271_2022_342_MOESM1_ESM.docx]

**Table S1: Consolidated criteria for reporting qualitative studies (COREQ): 32-item checklist checklist**

| Item/Topic | Guide questions/description | Reported in section |
| --- | --- | --- |
| Domain 1: Research team and reflexivity | | |
| Personal Characteristics | | |
| 1. Interviewer/facilitator | Which author/s conducted the interview or focus group? | Methods |
| 2. Credentials | What were the researcher's credentials? E.g. PhD, MD | Author list |
| 3. Occupation | What was their occupation at the time of the study? | Methods |
| 4. Gender | Was the researcher male or female? | Methods |
| 5. Experience and training | What experience or training did the researcher have? | Methods |
| Relationship with participants | | |
| 6. Relationship established | Was a relationship established prior to study commencement? | Methods |
| 7. Participant knowledge of interviewer | What did the participants know about the researcher? | Methods |
| 8. Interviewer characteristics | What characteristics were reported about the interviewer? | Methods |
| Domain 2: Study design | | |
| Theoretical framework | | |
| 9. Methodological orientation and theory | What methodological orientation was stated to underpin the study? e.g. discourse analysis, content analysis | Conceptual framework |
| Participant selection | |  |
| 10. Sampling | How were participants selected? e.g. purposive, snowball, etc. | Methods |
| 11.  Method of approach | How were participants approached? e.g. email, telephone, etc. | Methods |
| 12. Sample size | How many participants were in the study? | Results |
| 13. Non-participation | How many people refused to participate or dropped out? Reasons? | Results |
| Setting | | |
| 14. Setting of data collection | Where was the data collected? e.g. home, clinic, workplace | Methods |
| 15. Presence of non-participants | Was anyone else present besides the participants and researchers? | Methods |
| 16. Description of sample | What are the important characteristics of the sample? | Methods |
| Data collection | | |
| 17. Interview guide | Were questions provided by the authors? Was it pilot tested? | Methods |
| 18. Repeat interviews | Were repeat interviews carried out? If yes, how many? | No |
| 19. Audio/visual recording | Did the research use audio or visual recording to collect the data? | Methods |
| 20. Field notes | Were field notes made during or after the interview or focus group? | Methods |
| 21. Duration | What was the duration of the interviews or focus group? | Methods |
| 22. Data saturation | Was data saturation discussed? | Results |
| 23. Transcripts returned | Were transcripts returned to participants for comment or correction? | Methods |
| Domain 3: Analysis and findings | | |
| Data analysis | | |
| 24. Number of data coders | How many data coders coded the data? | Data analysis |
| 25. Description of the coding tree | Did authors provide a description of the coding tree? | Data analysis |
| 26. Derivation of themes | Were themes identified in advance or derived from the data? | Data analysis |
| 27. Software | What software, if applicable, was used to manage the data? | Data analysis |
| 28. Participant checking | Did participants provide feedback on the findings? | Methods |
| Reporting | | |
| 29. Quotations presented | Were participant quotations presented to illustrate themes/findings? Was each quotation identified, e.g. participant #? | Results |
| 30. Data and findings consistent | Was there consistency between the data and the findings? | Discussion |
| 31. Clarity of major themes | Were major themes clearly presented in the findings? | Results |
| 32. Clarity of minor themes | Is there a description of diverse cases or minor themes? | Results |

**Table S2: Interview Information Sheet**

November 2019

Lancet Commission on a synergistic approach to universal health coverage, health security and health promotion

This study is carried out by a team from the Norwegian Institute of Public Health and is part of the Lancet Synergies Commission that is investigating the challenges of fragmentation and potential of synergies across three global health agendas - universal health coverage (UHC), global health security (GHS), and health promotion (HS). The primary aim is to identify a set of key policies, institutional capacities, and interventions that will facilitate progress in individual agendas while amplifying progress in the others.

As part of this research, this project is focusing on how national public health institutes may contribute to creating synergies and addressing fragmentation across these agendas. The aim of this project is to explore how national public health institutes function in different countries and examine how their core functions contribute to synergies or fragmentation across these agendas. The work will also investigate key attributes of NPHIs such as national scope, autonomy, scientific basis, country focus, networking, accountability, and transparency.

We aim to provide insights and recommendations that are useful for governments, global health organizations, and academic institutions, and technical agencies. Outputs will reflect on what has worked, what has been problematic, and what strategies are of value to the global and national health communities.

We are inviting you to take part in the study by participating in an interview to capture your thoughts and experiences regarding national public health institutes and the global agendas as well as issues relating to synergies and fragmentation in low and middle-income countries. The interview will last no more than one hour. With your permission, we will take notes and record the interview.

Your name will not be used in any public documents. Data will be stored in a secure server in Oslo and may be shared with other authenticated researchers who are obligated by the same rules of confidentiality. If you prefer not to take part in this study, or if you would like to withdraw at any time, you are free to do so, without any consequences.

**Table S3: Informed Consent form**

Lancet Commission on a synergistic approach to universal health coverage, health security, and health promotion

**Please check all of the boxes that apply:**

| I have been given a clear overview of the study. |  |
| --- | --- |
| I understand that you will write about what I have said during our interview but will not reveal my identity in any study outputs. |  |
| I understand that the interview will be audiotaped. |  |
| I understand that you may include quotations from this interview in publications, reports, web pages and other research outputs. |  |
| I understand that information collected in interviews will be transferred to Norway. |  |
| I understand that the notes of this interview to be archived (anonymously) on a secure server at the Institute of Public Health. |  |
| I understand that notes from this interview may be shared with other authenticated researchers if they agree to preserve the confidentiality of information as noted in this form. |  |
| I understand that authenticated researchers may include quotations from this interview in publications, reports, web pages, and other research outputs if they agree to preserve the confidentiality of information as requested in this form. |  |
| I am willing to be interviewed. |  |
| **Interviewee Name (in block letters): Given the remote nature of this interview, by typing my name below, I am electronically signing this form.**  Signature: Date: | |
| **Researcher Name (in block letters):**  **Signature: Date:** | |

**Table S4: Interview Guide**

Interview guide questions

1. When was the NPHI/CDC established? Why was the NPHI established?
2. What is the scope of your NPHI? (i.e., national, regional, combination)?
3. How many staff work fulltime at the NPHI?
4. Does your NPHI have a legal foundation?
5. What core public health functions does your NPHI engage in? (i.e., surveillance, research, etc.)
6. How does your country’s NPHI contribute to universal health coverage (UHC), health security? (HS), and/or health promotion (HP)?
7. Has your NPHI had any experiences with bridging these global health agendas or facilitating synergies?
8. Do you think that NPHIs reduce or contribute to fragmentation across the health sector? If so, how?
9. Please describe how your NPHI (or agency/MoH) contributes to the following:
   1. Better coordination in the health sector?
   2. Stronger collaboration in the health sector?
   3. Policy coherence among these agendas?
   4. Alignment of national strategic plans or priorities? or competing priorities?
   5. Country ownership (in contrast to ownership by donors, external partners)
   6. How important is leadership?
10. What are critical success factors or lessons learned by your NPHI that may be useful for other NPHIs?
11. What is your NPHI’s long-term plan for sustainability?
12. Given the nature of public health challenges, what direction do you see for NPHIs and their role?
13. Do you have any recommendations for others that we should interview?
14. Do you have any final comments?

| Line | Key informant quote | Key informant ID # |
| --- | --- | --- |
| 1. | “*I don’t see them as distinct separate entities. There’s convergence and there’s overlap”* | #5 |
| 2. | *“…making investments that are focused on any of these [health security, health promotion, and universal health coverage], without that broader thinking about how these overlaps can be achieved is a waste”* | #1 |
| 3. | *“Public health problems need multisectoral and holistic interventions that involve a lot of actors and sectors not only the Ministry of Health or health care system. It’s crucial to address social determinants, environmental determinants, and political issues regarding health. We need institutions that are really cross-sectoral and can facilitate the implementation of public health policy”* | #10 |
| 4. | *“[NPHIs] are evidence-based and can use the data that they have to give credibility to policy decisions. The scientific credibility builds trust.”* | #16 |
| 5. | “*If we can collectively make sure that NPHIs are working on data, that will add value to the Ministry.”* | #14 |
| 6. | *“It's important that people understand that for the future and protection of the health of our people, the institute will play a role. It’s one of my jobs to make sure that the government puts more money on this type of thing but until now, they see it as a luxury.”* | #22 |
| 7. | *“To bring all these people together and come up with one plan where everybody feels like they own the plan.”* | #12 |
| 8. | *“The major challenge in running a lab is rarely the reagents. It’s the overhead, biosafety, biosecurity, infrastructure needs, power, electricity, water, internet. It’s just extremely inefficient to supply all that for just one disease so we have to build platforms rather than targeted interventions.”* | #11 |
| 9. | *“There are a lot of cost savings possible if you pool activities together so rather than having teams do HIV surveillance or TB surveillance or HCV surveillance, pool all these together into teams that are responsible for surveillance in general and build platforms that can be used and adopted to various infectious disease surveillance activities”* | #11 |

**Table S5 – Key informant quotes**
